# Prevalence of Hearing Loss and Perceptions of Hearing Health and Protection among Florida Firefighters

**DOI:** 10.3390/ijerph20053826

**Published:** 2023-02-21

**Authors:** Barbara Millet, Hillary A. Snapp, Suhrud M. Rajguru, Natasha Schaefer Solle

**Affiliations:** 1 Department of Interactive Media, University of Miami, Miami, FL 33136, USA; 2 Department of Otolaryngology, University of Miami, Miami, FL 33136, USA; 3 Department of Biomedical Engineering, University of Miami, Coral Gables, FL 33136, USA; 4 RestorEar Devices LLC, Bozeman, MT 59715, USA; 5 Department of Medicine, University of Miami, Miami, FL 33136, USA

**Keywords:** noise exposure, noise-induced hearing loss, hearing protection devices, occupational safety

## Abstract

Firefighters are exposed to extensive hazardous noise while on the job, both during routine tasks at the station and when responding to calls. However, little is known about firefighters’ occupational noise hazards. This study employed mixed methods, including focus groups, a survey, and audiometric testing, to identify sources of noise in the firefighters’ work environment, determine hearing protective strategies, discern firefighters’ perceptions of occupational noise exposure and impacts to their health, and quantify the prevalence of hearing loss among South Florida firefighters. A total of 6 senior officers served in an expert panel, 12 participated in focus groups, 300 completed the survey, and 214 received audiometric tests. Most firefighters were unaware of the risk and their departments’ policies, and did not participate in hearing protection practices and avoided using hearing protection devices, which they believed impede team communication and situational awareness. Nearly 30% of participating firefighters showed mild to profound hearing loss, a prevalence that is considerably worse than expected by normal aging alone. Educating firefighters about noise-induced hearing loss early in their careers may have significant health implications for their future. These findings provide insights for developing technologies and programs to mitigate the effects of noise exposure in the firefighting population.

## 1. Introduction

Recurrent or high levels of noise exposure have been shown to cause irreversible damaging effects to the inner ear, leading to noise-induced hearing loss (NIHL) and vestibular loss (NIVL). This damage is compounded over time and delayed in onset, and thus is typically not detected until it is permanent and severe. NIHL cannot be cured but it can be prevented by avoiding hazardous sounds or mitigating the impact of unavoidable hazardous sounds through hearing protection and environmental controls. 

NIHL has long been recognized as an occupational disease. Firefighters, who approximate 1.1 million of US workers, are exposed to recurrent hazardous noise both during routine tasks (e.g., equipment checks) at fire stations and when responding to emergency calls [1,2]. Approximately 54% of firefighters’ cumulative noise exposure exceeds the National Institute for Occupational Safety and Health’s (NIOSH) recommended exposure limit of 85 decibels over an 8 h period [1], putting them at increased risk of acquiring occupational hearing loss. Despite these recognized risks, little is known about firefighters’ perceptions of noise exposure and associated health outcomes, such as tinnitus, hearing loss, and balance problems (i.e., imbalance).

Firefighters’ risk for NIHL is heightened not only because of their repeated exposure to hazardous noise, but also because of their infrequent use of hearing protection devices (HPDs) [3,4]. Firefighters believe, with reason, that HPDs interfere with their ability to hear commands during emergency conditions and to use other required safety equipment [5]. Additionally, HPDs are often forgotten when gearing up [3]. The firefighters acknowledged the significance of good hearing in firefighting but perceived NIHL as an unavoidable consequence of the job and a small risk compared to other, more immediate hazards [3,5]. However, hearing impairment is not only a long-term consequence; in fact, NIHL is a significant problem for active firefighters, with many already reporting hearing difficulties [6,7,8] and other handicaps such as tinnitus in the fire service [4,9].

Beyond hearing loss, extended periods of hazardous noise exposure can also lead to a loss of balance, which poses a problem in most essential firefighting tasks. Increasing evidence demonstrates higher rates of balance impairment in other noise-exposed occupational groups, including military populations [10,11,12,13,14]. Furthermore, problems in the balance system may increase risk for cognitive decline [15].

Limited research has been conducted that focuses on both hearing and balance in the fire service. In addition, firefighters’ perceptions of hearing risks associated with their occupation have not been studied extensively. Therefore, this study provides new insight into the prevalence of hearing loss in the fire service and firefighters’ perceptions of both their noise exposure and their risk of hearing loss.

## 2. Methods

We employed mixed methods to (1) identify career firefighters’ perceptions of occupational noise exposure and its perceived health impact, (2) determine existing use of hearing protection devices, and (3) establish the prevalence of hearing loss. All subjects consented to participate, and all research activities complied with the American Psychological Association Code of Ethics and were approved by the Institutional Review Board at the University of Miami. No incentives were provided to the participants. We used organizational recruitment strategies to contact and recruit active firefighters in Florida. Working with established fire agency partners, we were able to share recruitment flyers through fire agency listservs and social media outlets. Organizational recruiting is an effective strategy when recruiting participants who are part of an occupational group. Participants were also recruited through convenience sampling, stemming from emails sent to organizational contacts and then expanded via snowball sampling [16]. However, to avoid self-selection bias, we also recruited participants at fire agency-led, compulsory, annual checkups, yielding 85% of the survey respondents and 100% of the hearing test participants.

### 2.1. Expert Panel

We conducted a meeting with fire service experts, consisting of six high-ranking officers in administration and safety, each with more than 20 years of experience, representing six South Florida fire agencies. The session was conducted at a fire department headquarters, with three experts attending in person and three remotely via video conferencing. At the meeting, we discussed firefighters’ hearing health and existing workplace policies and practices to reduce hazardous noise exposure. This session informed development of a survey and focus group discussion guide directed at high-risk firefighters. The meeting also helped establish a partnership between the participating fire agencies and our research group to address hearing health in the fire service.

### 2.2. Firefighter Focus Groups

We conducted four online focus group sessions with participants representing five Florida fire agencies using Zoom video conferencing software (version: 5.12.9). Of the 12 participating firefighters, three had already been diagnosed with hearing loss (25% positivity rate). The participants’ ranks included firefighters, drivers, captains, and chiefs, with a mean age of 49 ± 8.6 years and a mean of 22 ± 8.2 years of experience.

Following standard focus group procedures [17], we developed an outline to investigate the following topics: firefighters’ definition of noise and hazardous noise, sources of noise on scene and at the station, hearing protection use, and workplace policies for hearing conservation. Follow-up questions were asked, as needed, to explore topics in greater depth. Each focus group lasted approximately 60 min. 

### 2.3. Survey

We developed a 53-item survey, consisting of four categories: demographics (6 questions), occupational history (13), health history (23), and hazardous noise exposure in the workplace (11). Demographic data collected included respondent’s age, gender, race, ethnicity, marital status, and education. The occupational history category asked respondents about their current role in the fire service, total years of experience in the fire service, whether they had a second job, and if so, what type, and whether they had previously served in the armed forces, and if so, did they serve in a combat or war zone. The health history category inquired about respondents’ health coverage plan and history of cardiovascular disease, diabetes, and hearing problems. The hazardous noise exposure category asked about noise exposure at work, types of alarms heard at the station, duration of noise exposure during a shift, and symptoms after noise exposure, such as ringing in the ears or muffling of sounds, and/or suffering imbalance, disorientation, or vertigo (among other symptoms). Three-hundred firefighters completed the survey, including the 12 focus group participants. 

### 2.4. Hearing Thresholds

Prior to audiologic assessment, all participants received a visual examination of the ear canal and tympanic membrane to ensure normal anatomy and unobstructed, debris-free external canals. Hearing thresholds were measured using air-conducted pure-tones with noise-reduction insert headphones (ER-3A insert earphones, Etymotic Research Inc., Elk Grove Village, IL, USA) for both ears and an MA40 portable diagnostic audiometer (MAICO, MN, USA) calibrated to meet the American National Standards Institute standards. Hearing thresholds were obtained for test frequencies of 1, 2, 3, 4, 6, and 8 kHz in 5-decibel (dB) steps using a modified Hughson–Westlake procedure [18]. We included low frequencies (up to 3 kHz) for their importance in speech communication and high frequencies (4 to 6 kHz) that are typically impacted by hazardous noise. Normal hearing was defined as hearing thresholds of ≤25 dB for all the frequencies tested [19,20,21].

Audiologic measurements were collected onsite at local fire stations in southeast Florida. Trained research personnel performed measurements in an enclosed room that was free of interference from external noise. Noise levels were measured intermittently throughout testing to meet the ANSI/ASA S3.1-1999 (R2018) standard for maximum permissible ambient noise levels, not exceeding 45 A-weighted decibels (dBA), for audiometric testing [22,23]. A limitation of this study is that participants’ baseline audiograms were not accessible.

### 2.5. Data Analysis

We transcribed audio recordings of each focus group, and then two members of the research team analyzed all transcripts through an inductive approach to identify themes and concepts. Open-ended responses from the survey were reviewed and categorized using combinations of common strings. All categorizations were made following a consensus approach. Descriptive statistics were analyzed for the survey variables and hearing test results. Means and standard deviations for continuous variables, such as age, were calculated. Values for categorical variables were summarized as frequencies and percentages. For the survey, Pearson correlation coefficients were calculated for ordinal and continuous variables and HPD use. Chi-square analysis was conducted to explore associations between dichotomous variables and HPD use. To identify factors that influence HPD use, we conducted stepwise linear regression, where independent variables were initially entered into the multiple regression to determine their collective contribution to HPD use. The independent variables without a substantial contribution (*p* > 0.10) were eliminated sequentially until the best predictors of HPD use were identified. The coefficients of determination (R^2^) were obtained for each entry to determine the variables’ contribution to HPD use. We computed standardized regression coefficients to equalize measurements using different scales that allow us to determine which independent variables had the greatest effects on HPD use. We also assessed the assumptions of normality of the residuals and heteroscedasticity.

We explored mean differences of Hearing Threshold Levels (HTLs) between ears using paired-samples t-test. Analysis of Covariances (ANCOVAs) was used to calculate differences in means of HTLs at low and high frequencies by years of experience in the fire service, while controlling for age. We used Mann–Whitney analyses to examine differences in the prevalence of hearing loss at low frequencies (1 to 3 kHz) and high frequencies (4 and 6 kHz) between groups. A *p*-value of 0.05 or less was considered statistically significant. Quantitative data analyses were performed using the SPSS^®^ software (version 28.0; New York, NY, USA: IBM Corp^®^).

## 3. Results

### 3.1. Expert Panel

The qualitative analysis revealed five major themes for understanding NIHL in the fire service. The themes included the following: (1) the lack of understanding of the damage caused by noise exposure, (2) the barriers to hearing protection use, (3) the perceived sources of hazardous noise, (4) the inadequacy in current hearing protective policies and programs, and (5) the recommendations for future hearing conservation programs. The quotations provided are representative samples of the overarching themes.

#### 3.1.1. Lack of Understanding of the Damage Caused by Noise Exposure

Panelists reported that firefighters generally do not perceive loud noises in their work environment to be hazardous and that they are unaware of the varying sources of loud noise in the fire service. The expert panel explained that the lack of understanding of hazardous noises at work is more prevalent for early career firefighters. Mid-to-late career firefighters become aware only after experiencing hearing loss. Even though the participating fire agencies require annual screening, the panel indicated that screening alone was insufficient to reduce the risk of hearing loss. 

#### 3.1.2. Barriers to Hearing Protection Use

The expert panel explained that firefighters’ inability to communicate with their teams and the loss of situational awareness were the greatest barriers to HPD use. Heightened situational awareness was deemed critical for safe operations on scene. Therefore, the panel recommended that researchers should focus on hearing conservation programs for routine tasks at the station as the greatest opportunity to impact cumulative noise exposure as firefighters spend a considerable amount of their shift at the station.

*“Eliminating a sense or masking a sense goes against everything we teach them.”*

*“I’d rather be deaf than dead.”*

Other reasons given for not using HPDs included lack of availability, not being able to use HPDs comfortably when available, and peer pressure.

*“Someone who wants to wear hearing protection may not do so because others on the engine aren’t doing it.”*

#### 3.1.3. Perceived Sources of Hazardous Noise

The panel listed the sources that they believed to be damaging to hearing, such as noises from hydraulic pumps, helicopters, gas leaks, and two-way radios. They also stated that drivers have right ear hearing deficiencies because of the motor’s location on the fire engine. On a day-to-day basis, two-way radios, as well as station alerting systems, are usually set at the highest volume (given the lack of global safety standards for these systems).

*“The radio is always set loud and then you put it right on your ear because you can’t hear the radio with all the background noise.”*

*“Even if you are not going on the call, you will be notified that a call is going on.”*

#### 3.1.4. Inadequacy in Current Hearing Protective Policies and Programs

The experts provided mixed responses regarding policies for hearing protection use. Three of the six panelists indicated having policies recommending the use of HPDs in morning equipment checks. The other panelists were unsure of their agencies’ recommendations for HPD use and indicated that any current policy was mostly informal and barely followed. Furthermore, all panelists indicated that the only real effort initiated was to include hearing screening with the annual physical exam. 

#### 3.1.5. Recommendations for Future Hearing Conservation Programs

Experts suggested implementing research on improving hearing health and training programs to increase awareness of noise risks. They identified three important actions: (1) collecting fire station noise measurements, (2) gathering evidence of progressive change to firefighter hearing, and (3) educating firefighters about NIHL early in their career. These findings provide insight into developing programs to mitigate the effects of firefighters’ noise exposure. 

### 3.2. Firefighter Focus Groups

The qualitative analysis of the firefighter focus groups revealed three major themes: (1) sources of hazardous noise exposure, (2) health consequences of hearing loss, and (3) barriers for hearing protection use.

#### 3.2.1. Sources of Hazardous Noise on the Job

Firefighters indicated considerable noise level variability depending on the type of activity and activity location. The identified sources of the noise were unique to the fire service and included riding fire and emergency trucks with and without active sirens; operating water pumps and power tools; daily checks of personal alert safety system alarms; opening and closing bay doors; and having to shout to communicate over their radios and other environmental noise. The firefighters stated that the loudest noise exposure occurred on scene, with many competing sources of noise in the background at variable levels, while having to attend to and respond to radio and in-person communications.

#### 3.2.2. Effects of Hazardous Noise on Health

When asked to describe the effects of hazardous noise exposure, the participants indicated the following: suffering from ringing or buzzing in the ear, disruption of effective communication, and impaired hearing. Across the focus groups, the firefighters perceived hearing loss as an unavoidable consequence of the job. Conversely, the participants were unaware of how noise exposure may impact other aspects of health, including balance [14] and cognitive decline [24]. Many participants stated that if they did have issues with balance, they would not report it as it may result in changes to their job assignments.

*“Balance issues would get you kicked off the truck.”*

#### 3.2.3. Barriers to Hearing Protection Device Use

All participants indicated that no formal training was provided and that no standard operating policies were in place for hearing protection use. Participants also indicated that not wearing HPDs during routine activities such as morning equipment checks posed unnecessary risks to their hearing health. However, HPDs are often not used in routine daily activities because they are generally forgotten or unavailable. Another reason was not being able to detect a threat from behind, such as a perpetrator attack. Perceptions of HPD use by the focus group participants aligned with those reported by the expert panel, that HPDs posed dangerous risks to firefighters and reduced their situational awareness on scene. Participants expressed a preference for integrating communications with safety equipment and incorporating technology that dampens hazardous noise while highlighting radio communication.

*“If you put stuff in your ears to protect your hearing, you are not going to hear the things you need to hear.”*

### 3.3. Survey

#### 3.3.1. Participant Demographics and Health History 

A total of 300 firefighters representing 16 Florida fire agencies completed the survey. The mean age of the respondents was 39.1 (*SD* = 9.9) years, with 94% male and 6% female participants. Most (83%) identified as white, 66% were Hispanic, and 96% were college graduates or had completed some college courses. Many of the participants (90%) were career firefighters with a mean tenure of 14 years on the job (*SD* = 8.6). Service ranks included firefighters (32%), driver operators (20%), investigators (1%), lieutenants (15%), captains (12%), and chiefs (9%). Firefighters reported responding to eight or more calls on average per shift. A few of the participating firefighters (21%) reported having a second job while 17% reported having prior military experience. The firefighters were in overall good health with only 2% reporting a history of cardiac disease and 2% having diabetes. The socio-demographic and employment characteristics of the participating firefighters are outlined in Table 1.

#### 3.3.2. Hearing and Vestibular Health 

When asking firefighters about their perception of hearing loss risk, 74% said that they were not concerned about their hearing at the time of the study, whereas 82% of the same participants reported having a slight to extreme concern about hearing loss in the future. Nineteen percent of participants reported having seen an ear, nose, and throat specialist (ENT) regarding hearing damage symptoms. The most reported reasons for seeing an ENT were ear infections (8%), followed by ringing in the ear (6%), and hearing loss (5%). Ten percent reported being diagnosed with a hearing problem, with 4% having had hearing surgery on both ears, and 3% reporting use of hearing aids. Regarding vestibular health, 74% of respondents were not concerned about losing their sense of balance in the future. Three percent reported being diagnosed with vertigo.

#### 3.3.3. Hazardous Noise Perceptions and Exposure Symptoms

**Figure 1:**
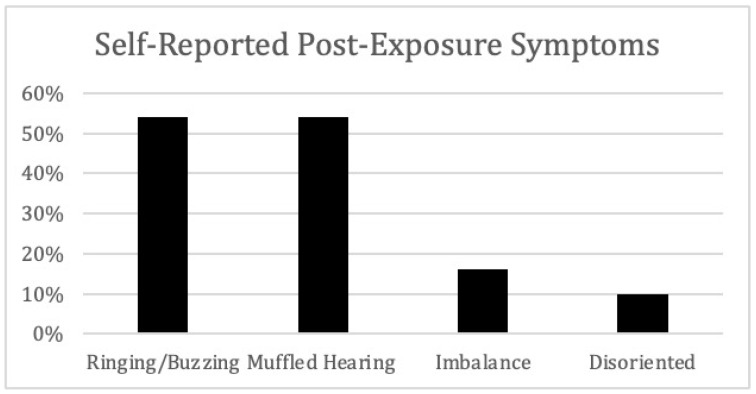
Percent of self-reported post exposure symptoms.

Eighty-two percent of participating firefighters reported exposure to hazardous noise at work, with 66% reporting hazardous noise exposure occurring ‘occasionally’ to ‘frequently’ in a typical work shift. Fifty-four percent of participants reported having ringing or buzzing in their ears (see Figure 1). Additionally, 54% of respondents reported muffled hearing, 16% reported experiencing imbalance, and 10% of respondents reported having felt disoriented after exposure to hazardous noise.

#### 3.3.4. Hearing Conservation Practices on the Job 

**Figure 2:**
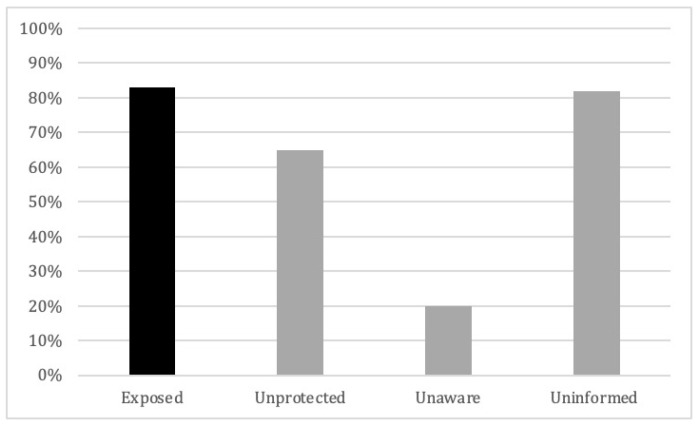
Percent self-reporting noise exposure at work (Exposed) and hearing protection usage (Unprotected), awareness of existing workplace hearing protection policies (Unaware), and knowledge of NIHL (Uninformed.)

For the agencies included in our study, hearing screening is mandatory at recruitment and during firefighters’ annual physical. Even so, only 59% of participants reported ever having had a hearing test, indicating that firefighters may not be aware that their hearing was being tested. Of those who reported having received a hearing test, the majority (83%) cited employment-mandated routine care as the reason. Other reasons for taking a hearing test included the following: ringing in the ear, muffled hearing, changes to hearing, and feeling off-balance. Remarkably, 82% of all participants reported never having received information about NIHL at work, and 20% were unaware of work policies or recommendations for hearing protection use (see Figure 2). In our sample, 83% reported exposure to hazardous sounds during a work shift, yet 65% reported ‘never’ to ‘rarely’ wearing HPDs at work. Of those reporting HPD use, 6% reported having a hearing loss diagnosis.

Table 2 shows the correlation among several variables studied. The highest correlation coefficient was observed between age and years worked (*r* = 0.88, *p* < 0.01), and both were significantly, positively correlated (all at *p* < 0.01) with HPD use. Individuals tend to wear HPDs more frequently once they have already been diagnosed with hearing loss [25,26]; therefore, as expected, hearing damage symptom variables significantly correlated with HPD use (*p* < 0.05). Notably, the highest correlation coefficient for HPD use was with frequency of ringing in the ear after hazardous noise exposure (*r* = 0.24, *p* < 0.01), in that participants who reported greater HPD use at work also indicated greater occurrences of their symptoms.

There were also significant associations between HPD use and hearing loss diagnosis ($\chi^{2}\left(4\right)$ = 9.64, *p* < 0.05), and HPD use and firefighters’ current concerns about their hearing ($\chi^{2}\left(4\right)$ = 18.38, *p* = 0.008). Greater HPD use was observed in firefighters who had been previously diagnosed with hearing loss and firefighters who expressed current concerns about their hearing. However, the association between future concerns about hearing loss and HPD use was not significant ($\chi^{2}\left(4\right)$ = 4.21, *p* = 0.378). HPD use was also associated with work recommendations ($\chi^{2}\left(4\right)$ = 39.50, *p* < 0.001), in that, participants who reported greater HPD use at work also indicated that their work recommended HPD use on the job. Furthermore, perceptions of a hazardous noise work environment were significantly related to HPD use, $\chi^{2}\left(4\right)$ = 29.92, *p* < 0.001, such that firefighters who reported ‘occasional’ to ‘frequent’ hazardous noise exposure at work were also reporting greater HPDs use. 

Stepwise linear regression with backward elimination was conducted to investigate factors influencing HPD use. Based on the correlational and univariate analyses, factors showing significant associations with HPD use were included in the regression model as independent variables: firefighter length of service, number of hearing damage symptoms, frequency of ringing in the ear and muffled hearing, current and future hearing concerns, hearing loss diagnosis, perceptions of hazardous noise exposure at work, perceived duration of hazardous noise exposure at work, having received information about NIHL at work, and awareness of work policies or recommendations for hearing protection use. HPD use was included as the dependent variable. 

Table 3 presents the results of the stepwise linear regression, which demonstrates that years of experience in the fire service (*β* = 0.035, *p* = 0.021), number of hearing damage symptoms (*β* = 0.502, *p* = 0.003), and awareness of work policies or recommendations for hearing protection use (*β* = 0.930, *p* = 0.008) had significant and positive associations with HPD use. According to the coefficients and *p*-values, and when controlling for the other factors, significant differences were observed in HPD use: HPD use increased significantly with years in the fire service, hearing damage symptoms, and awareness of employer recommendations for HPD use. 

### 3.4. Hearing Loss

Two-hundred and fourteen of the 300 survey respondents participated in the hearing test. Table 4 presents the means and standard deviations of HTLs for the left, right, and worst ear, the mean difference between the left and right ears, and the prevalence of hearing loss at each of the tested frequencies. We found no statistically significant differences in hearing ability between the left and right ears, even at the higher frequencies. In both ears, the greatest mean HTLs occurred at the higher frequencies (4, 6, and 8 kHz). 

Table 5 shows the distribution of hearing loss at the low and high frequencies. The means of HTLs for the worst ear at the low frequencies (1, 2, and 3 kHz combined) and the noise-sensitive, high frequencies (4 and 6 kHz combined) were 23.9 and 26.1, respectively. About 15% of firefighters showed mild to severe hearing loss at the low frequencies, and 23% showed mild to profound hearing loss at the high frequencies. Some firefighters (9%), all with 10 or more years in the fire service, had moderate to profound hearing loss at the noise-sensitive frequencies (4 and 6 kHz); 2% had moderate to severe hearing loss at the low frequencies. All participants with hearing loss at the lower frequencies also had hearing loss at the higher frequencies.

#### 3.4.1. Hearing Loss and Fire Service Experience

**Figure 3:**
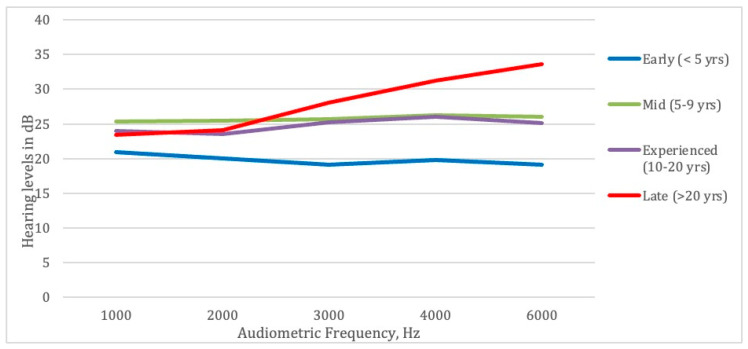
HTLs in worst ear at all tested frequencies by years worked in the fire service.

Participants were classified into four experience groups by years of service: (1) early career defined as less than 5 years in the fire service, (2) mid-career defined as 5–9 years, (3) experienced defined as 10–20 years, and (4) late career defined as those with 20 or more years of service. Figure 3 shows the mean HTLs in the worst ear at each tested frequency by years of service. HTLs increased with the greater time served. The figure also shows that HTLs increase more at the noise-sensitive frequencies, even for mid-career and experienced firefighters, and dramatically increase for late-career firefighters. 

We conducted an ANCOVA to explore the relationship between HTLs and years in the fire service, while controlling for age. As previously noted, age and years in the fire service were positively, highly correlated (*r* = 0.88). However, the variance inflation factor (VIF) between age and years in the fire service for our model was 1.010, which is below the concern of collinearity raised by VIFs of 10 or above [27]. The results indicate a significant main effect of years of service on HTLs at both low frequencies (*F*(3198) = 5.279, *p* = 0.002) and noise-sensitive, high frequencies (*F*(3198) = 2.661, *p* < 0.05), after controlling for age. The covariate, age, was significantly related to HTLs at the high frequencies (*F*(1198) = 8.996, *p* = 0.003), but not significant at the low frequencies (*F*(1198) = 1.281, *p* = 0.259). Pairwise comparisons indicate significant differences in HTLs at the low frequencies between early and mid-career firefighters (*p* = 0.001). Bonferroni correction was applied; thus, the effects are reported at a 0.008 level of significance.

#### 3.4.2. Hearing Loss and Concern about Hearing Loss 

Mann–Whitney tests were conducted to compare HTLs for the worst ear at low and high frequencies on the firefighters’ self-reported levels of concern about hearing loss. The HTLs at the low frequencies showed significant differences between those with and without current concerns about hearing loss (U = 4016, *z* = 2.014, and *p* = 0.044). HTLs at the noise-sensitive, high frequencies also showed statistically significant differences between the groups, with firefighters having more severe hearing loss showing greater concern (U = 4663, *z* = 4.132, and *p* < 0.001).

#### 3.4.3. Hearing Loss and Symptoms

Mann–Whitney tests were conducted to compare HTLs, for the worst ear at low and high frequencies, to reports of any hearing damage symptoms. HTLs at low frequencies did not differ significantly between the two groups (i.e., those with and without hearing damage symptoms), U = 5292, *z* = 1.811, *ns*. However, at the noise-sensitive, high frequencies, the firefighters reporting hearing damage symptoms had more severe hearing loss, U = 5910, *z* = 3.478, and *p* < 0.001.

#### 3.4.4. Hearing Loss and HPD Use

Mann–Whitney tests showed that there was a statistically significant association of HPD use and HTLs at both low frequencies (U = 2405, *z* = −2.151, and *p* = 0.031) and at the noise-sensitive, high frequencies (U = 3419, *z* = −1.982, and *p* = 0.047). Moreover, firefighters with hearing loss at the low frequencies, who reported greater HPD use, also had more severe hearing loss at the noise-sensitive, high frequencies.

## 4. Discussion

Research indicates that firefighters experience an elevated risk of developing hearing loss. Our results demonstrated that firefighters underestimate noise hazards in the workplace; most already experience hearing damage symptoms and some already have hearing loss, and a significant portion do not adequately protect their hearing. We found that firefighters can identify some but not all sources of hazardous occupational noises, often underestimating the noise levels or potential sources of noise hazards. The underestimation of noise levels may also be due to firefighters becoming accustomed to noise. Reduced awareness of hazardous noise risk was particularly evident for routine activities at the station, as opposed to exposure in emergency responses. Participants were also generally unaware of the relationship between sound levels and duration of exposure to hazardous noise. Specifically, when asked about occupational factors that contribute to hearing loss, none of the study groups, including the expert panelists, focus group participants, or surveyed firefighters, identified duration of exposure as a factor of concern. Most participating firefighters understood that repeated exposure to loud noises increases the risk of hearing loss later in life, yet they expressed limited concern about the current risks of hearing loss. 

Despite their lack of concern, 10% of our firefighter sample reported having already been diagnosed with hearing loss (mean age 45 years). Alarmingly, 58% of the participating firefighters reported other damage to the ear. Many (54%) reported having muffled hearing after noise exposure, a sign of a temporary threshold shift that often occurs following exposure to hazardous noise. Although threshold shifts often return to normal, evidence demonstrates that permanent damage to the inner ear has occurred and is linked to the early onset of hearing loss [28,29,30,31]. In addition, 54% reported ringing or buzzing in the ear, otherwise known as tinnitus, another known marker of inner ear damage. 

Even though only 10% reported a diagnosis of hearing loss, the results of the hearing test show that, in fact, nearly 30% of the firefighters in this study showed at least mild hearing loss across low and high frequencies, indicating that firefighters may not be aware of their own hearing loss. For our analysis, we used the WHOs hearing grading system [21], however, more recent hearing grading systems have promoted reducing the criteria of impairment from 26 to 20 dB HL [32,33]. When recalculating with a more contemporary scale [33], 134 participating firefighters in the previously categorized “normal hearing” group would be recategorized as having mild hearing impairment, increasing the percentage of detected hearing loss from 29% to 80%. While a less conservative grading system may facilitate earlier detection of changes in hearing function, nevertheless, determining which factors contribute to changes in hearing function for this occupational group remains a challenge. Specifically, the challenge lies in assessing to what extent early, mild hearing loss is related to auditory aging, environmental exposures, or a combination of aging with hazardous exposures. While the influences of aging and noise exposure on hearing loss are an increasingly recognized phenomena [34], the interaction of aging and noise on human hearing remains difficult to characterize and detect in the early stages.

In our study, firefighters reported a hearing damage and loss greater than expected by aging alone [35,36,37]. The participating firefighters’ positivity rates are far greater than that of the general population, in which 6.5% of individuals 40–45 years old have hearing loss [38]. Consistent with prior work, we found that firefighter hearing loss severity accelerates with increased years of service [3,6,12]. We also found that firefighters with hearing loss at the noise-sensitive frequencies also reported having hearing damage symptoms and were more concerned about their overall hearing health.

Studies have shown that adults with NIHL are more likely to experience falls [14,31,39,40] and that damage to the inner ear balance organ is associated with a greater risk of cognitive decline [41]. In our study, the firefighters were unfamiliar with the impact of noise exposure on other aspects of health, including balance and cognitive decline. None of the firefighters in our cohort reported any issues or concerns about changes in balance. They also indicated no knowledge of balance issues among their colleagues in the fire service. Additionally, we confirmed that firefighters are not aware that noise exposure impacts their vestibular system, which may be the cause of balance impairment. Studies have shown that adults with NIHL are more likely to experience falls [14,39], and that the prevalence of both hearing loss and imbalance increases with increasing age [15]. Recent work has shown that damage to the inner ear balance organ also increases fall risk and has been associated with a greater risk of cognitive decline [15]. Future studies should explore cognitive decline and balance impairment following noise exposure for this occupational group.

Noise-induced ear damage can be prevented by wearing HPDs. Thus, operational duties in the military, fire service, and other occupations with hazardous noise exposure often require the use of HPDs. However, participating firefighters reported rarely using their HPDs, noting valid ancillary safety concerns. This is consistent with findings showing that military personnel were concerned about using HPDs because of reductions in situational awareness, interference in detection and localization of auditory cues, and because of incompatibility with other military gear [42]. Furthermore, recent evidence demonstrated that the use of HPDs does disrupt spatial hearing, specifically in localization performance and in monitoring speech signals [43]. Overall, HPDs are often avoided because they cause an increase in listening effort, variability in sound-localization ability, and reduction of effective communication [42,43,44]. Adoption and acceptance of HPDs in the fire service continue to be low [45], despite longstanding efforts to address hazardous noise in occupational groups [1,46,47] and, in particular, the fire service [48,49,50]. Our findings indicated that firefighters are indeed concerned about the possibility of hearing loss in the future but do not see it as a priority, especially in comparison to the immediate risks caused by reduced operational readiness. As a result, most firefighters are not willing to take preventive actions, even when offered.

The National Fire Protection Association (NFPA) standards for safety and health recommend that fire departments create hearing conservation programs that reduce or eliminate harmful sources of noise and require hearing protection when noise exceeds 90 decibels [48,49]. The NFPA also recommends that firefighters complete a hearing exam at entry and periodically throughout their careers. Further, NFPA standards 1500 [49] and 1582 [48] recommend that hearing policies should be implemented per department, and that department-specific policies should be established. Despite these recommendations, our findings show that only 59% of the sample reported having a hearing exam and, what is more concerning is that 20% were unaware of workplace policies for hearing protection. These findings indicate that hearing protection and surveillance policies may not be fully implemented into practice, suggesting that hearing health is not sufficiently prioritized in the fire service. As a future direction, we recommend that policies related to hearing health and hearing protection be shared on a regular basis with members of the fire service. This type of teaching methodology has been successful with this population for other health initiatives, such as cancer prevention strategies [51]. Oftentimes, policies are missed if sent passively to the membership (i.e., emails or memos), but the integration of policies through training has been more successful [52]. Our findings offer insight into developing programs to mitigate the effects of noise exposure on the firefighting population; however, much more data need to be gathered. 

A limitation of the current study is that we did not collect noise exposure profiles of the firefighters’ work environments and across job categories in the fire service. Future studies should characterize firefighters’ noise exposure by location (stations and scenes) and across job roles and activities. Furthermore, firefighter’s work environments and hearing health need to be monitored longitudinally to better understand the frequency, duration, and hazardous levels of their noise exposure, which can inform the development of mitigation strategies and allow for better estimates of hearing health risks [53].

Firefighters’ substantial exposure to noise and associated health risks reveal a need for policies aimed at mitigating hearing health risks. Efforts are needed to integrate hearing-protective mechanisms with communication equipment and to implement more effective hazardous noise alerting systems and environmental controls. Mechanisms and controls must be widely accessible, easy-to-use, and accurate, while not compromising firefighter safety or performance. Additionally, it is important to provide training programs regarding NIHL, balance dysfunction, and the long-term impacts of hazardous noise exposure. This information, along with instructions on effective HPD use, should be included as part of the minimum standards training. Educating firefighters on these risks early in their career will likely benefit their overall hearing health. Research shows that informing workers of the health risks at entry level can change their behaviors, specifically related to risk avoidance [51]. Therefore, early training and policy changes can make a substantial impact on the behaviors of firefighters and the overall culture around hearing health.

## 5. Conclusions

Our findings indicate that firefighters are at considerable risk of NIHL. Efforts are needed to minimize the incidence of hearing loss and tinnitus in firefighters. A necessary next step is to increase awareness of factors affecting hearing health and to support health-promoting behaviors—specifically, the use of HPDs and other protective strategies—when using protection does not negatively impact firefighter safety and performance. Future studies are needed to better characterize noise in the work environment and to better understand the impact of using HPDs on firefighters’ situational awareness and job performance.

## Figures and Tables

**Table 1:** Socio-demographic and work characteristics of the participating firefighters.

Total Sample, *n* = 300	
Characteristics	Mean (SD)
**Age (years)**	: 39.1 (9.9)
**Tenure**	: 14.0 (8.6)
	**N (%)**
**Gender**	
Male	: 281 (93.7)
Female	: 19 (6.3)
**Ethnicity**	
Hispanic	: 197 (65.7)
Non-Hispanic	: 98 (32.7)
Unknown	: 4 (1.3)
**Race**	
Caucasian	: 249 (83.0)
African American	: 18 (6.0)
Other	: 18 (6.0)
Unknown	: 10 (3.3)
**Marital Status**	
Married	: 173 (57.7)
Divorced	: 27 (9.0)
Widowed:	1 (0.3)
Never married	: 71 (23.7)
Member of an unmarried couple	: 28 (9.3)
**Education Level**	
College graduate	: 112 (37.3)
Some college	: 174 (58.0)
High school graduate or GED	: 11 (3.7)
Some high school:	1 (0.3)
**Rank**	
Firefighter	: 97 (32.3)
Driver	: 59 (19.7)
Investigator	: 3 (1.0)
Lieutenant	: 45 (15.0)
Captain	: 37 (12.3)
Chief	: 27 (9.0)
**Second Job**	
Yes	: 63 (21.0)
No	: 198 (66.0)
**Military History**	
No, never on active duty except basic training	: 11 (3.7)
No, never served	: 247 (82.3)
Yes, now on active duty	: 2 (0.7)
Yes, active duty in the past	: 39 (13.0)

**Table 2:** Descriptive statistics and correlation coefficients for survey variables.

			Correlation Coefficient
Variables	Mean	SD	HPD Use
Tenure	14.0	8.6	0.178 **
Calls/shift	7.8	3.5	0.045
Hazardous Noise Exposure Duration	-	-	0.161 **
# of Symptoms	1.5	0.8	0.192 *
Freq. of ringing in the ear	-	-	0.237 **
Freq. of muffled hearing	-	-	0.191 **

* *p* < 0.05, ** *p* < 0.01.

**Table 3:** Coefficients of determination in the initial and best predictive models for HPD use.

				Residual Statistics	
Model	R	R^2^	R^2^ Change	F	P
Initial ^a^	0.625	0.390	0.390	2.935	0.003
Best ^b^	0.439	0.193	−0.197	7.887	<0.001

Dependent variable: HPD use. a Predictors: tenure, HL diagnosis, current HL concerns, future HL concerns, ringing in the ear freq., muffled hearing freq., calls per shift, hazardous noise exposure duration, count of hearing damage symptoms, HPD recommended, and informed about NIHL. b Predictors: tenure, HPD recommended, and count of hearing damage symptoms.

**Table 4:** Mean and standard deviation of HTLs and prevalence of hearing loss at all test frequencies (N = 214).

	Test Frequency (Hz)					
HTLs, dB	1 K	2 K	3 K	4 K	6 K	8 K
Left ear, mean (SD)	22.8 (7.5)	23.0 (8.3)	23.9 (10.8)	24.9 (12.3)	24.9 (11.6)	24.5 (10.8)
Right ear, mean (SD)	22.9 (6.2)	22.5 (7.1)	23.4 (9.0)	24.2 (10.9)	24.2 (11.4)	24.2 (11.7)
Mean difference between two ears, *t*-test (*p* value)	−0.4 (0.717)	1.6 (0.107)	1.0 (0.318)	1.3 (0.179)	1.3 (0.195)	0.6 (0.535)
Worst ear, mean (SD)	23.5 (7.3)	23.4 (7.8)	24.8 (10.3)	26.0 (12.3)	26.2 (12.5)	25.7 (12.3)
Hearing loss (HTL > 25 dB), N (%)	11 (5.1)	12 (5.6)	31 (14.5)	42 (19.6)	36 (16.8)	25 (11.7)

**Table 5:** Distribution of hearing loss, for the worst ear, at low (1, 2, and 3 kHz) and high (4 and 6 kHz) frequencies.

Hearing Loss Prevalence	1, 2, and 3 kHz, *n* (%)	4 and 6 kHz, *n* (%)
Normal (≤25)	181 (84.6)	165 (77.1)
Hearing loss		
Mild (26–40 dB)	28 (13.1)	29 (13.6)
Moderate (41–60 dB)	3 (1.4)	17 (7.9)
Severe (61–80 dB)	2 (0.9)	2 (0.9)
Profound (>80 dB)	0 (0)	1 (0.5)
Mean ± SD (Range)	23.9 ± 7.6 (5–65)	26.1 ± 11.8 (−2.5–92.5)

## Data Availability

Derived data supporting the findings of this study are available from the corresponding author (BM) upon request.
